# A Phenotypic Screen in Zebrafish Identifies a Novel Small-Molecule Inducer of Ectopic Tail Formation Suggestive of Alterations in Non-Canonical Wnt/PCP Signaling

**DOI:** 10.1371/journal.pone.0083293

**Published:** 2013-12-11

**Authors:** Evelien Gebruers, María Lorena Cordero-Maldonado, Alexander I. Gray, Carol Clements, Alan L. Harvey, Ruangelie Edrada-Ebel, Peter A. M. de Witte, Alexander D. Crawford, Camila V. Esguerra

**Affiliations:** 1 Laboratory for Molecular Biodiscovery, Department of Pharmaceutical and Pharmacological Sciences, University of Leuven, Leuven, Belgium; 2 Faculty of Chemistry Sciences, School of Biochemistry and Pharmacy, University of Cuenca, Cuenca, Ecuador; 3 Strathclyde Institute of Pharmacy and Biomedical Sciences, University of Strathclyde, Glasgow, Scotland; 4 Chemical Biology Group, Luxembourg Centre for Systems Biomedicine, University of Luxembourg, Esch-sur-Alzette, Luxembourg; New York Medical College, United States of America

## Abstract

Zebrafish have recently emerged as an attractive model for the *in vivo* bioassay-guided isolation and characterization of pharmacologically active small molecules of natural origin. We carried out a zebrafish-based phenotypic screen of over 3000 plant-derived secondary metabolite extracts with the goal of identifying novel small-molecule modulators of the BMP and Wnt signaling pathways. One of the bioactive plant extracts identified in this screen – *Jasminum gilgianum*, an Oleaceae species native to Papua New Guinea – induced ectopic tails during zebrafish embryonic development. As ectopic tail formation occurs when BMP or non-canonical Wnt signaling is inhibited during the tail protrusion process, we suspected a constituent of this extract to act as a modulator of these pathways. A bioassay-guided isolation was carried out on the basis of this zebrafish phenotype, identifying para-coumaric acid methyl ester (pCAME) as the active compound. We then performed an in-depth phenotypic analysis of pCAME-treated zebrafish embryos, including a tissue-specific marker analysis of the secondary tails. We found pCAME to synergize with the BMP-inhibitors dorsomorphin and LDN-193189 in inducing ectopic tails, and causing convergence-extension defects in compound-treated embryos. These results indicate that pCAME may interfere with non-canonical Wnt signaling. Inhibition of Jnk, a downstream target of Wnt/PCP signaling (via morpholino antisense knockdown and pharmacological inhibition with the kinase inhibitor SP600125) phenocopied pCAME-treated embryos. However, immunoblotting experiments revealed pCAME to not directly inhibit Jnk-mediated phosphorylation of c-Jun, suggesting additional targets of SP600125, and/or other pathways, as possibly being involved in the ectopic tail formation activity of pCAME. Further investigation of pCAME’s mechanism of action will help determine this compound’s pharmacological utility.

## Introduction

Chemical genetics in zebrafish enables [1] the identification of bioactive small molecules based on their ability to induce specific developmental dysmorphologies or behaviors [2], the association of *in vivo* phenotypes induced by these compounds with the modulation of key signaling pathways, and [3] ultimately, the elucidation of their biological targets. Because of multiple genetic screens carried out to date, a wide range of mutant phenotypes in zebrafish have been thoroughly characterized and reported, including for genes and pathway components of the BMP (Bone Morphogenetic Protein) and Wnt (Wingless/Int) signaling pathways. Dorsoventral (DV) and anterior-posterior (AP) axis defects as well as ectopic tail formation have been reported in BMP/Wnt mutant or transgenic zebrafish [4–7]. The recent identification of dorsomorphin, a novel small-molecule inhibitor of BMP signaling, proved the usefulness of phenotype-based compound screening in zebrafish embryos. This molecule induced severe dorsalization (DV patterning defect) in developing embryos [8] and to date this molecule and some of its derivatives have been extensively studied as modifiers of iron homeostasis, bone formation and metabolism [9]. Despite progress in the field, target elucidation remains the major challenge in the field of chemical genetics [10].

Over the last decade, zebrafish have proven increasingly useful as an animal model in the early drug discovery process [11,12]. For example, PGE2 was identified in a zebrafish screen for small molecules capable of causing the proliferation of hematopoietic stem cells (HSCs) *in vivo* [13], and is now in clinical trials as part of a novel HSC transplantation protocol. Using zebrafish for early-stage drug discovery has several key advantages. Zebrafish are highly fecund, rapidly develop *ex utero* and require simple husbandry. They are small, so only sub-milligram amounts of compounds are needed for screening. Their optical transparency permits live visualization using standard light microscopy. All this features have made this model very cost-efficient to use [11]. Furthermore, established genetic tools such as antisense morpholino oligonucleotides and targeted mutations via TALENs allow for relatively rapid disease modeling [14,15].

We and others have recently established zebrafish as a platform for natural product discovery [2,16] using bioassay-guided fractionation of secondary metabolite extracts to identify plant-derived small molecules with anti-angiogenic [17], anti-epileptic [3], and anti-inflammatory [1] activity. Molecules that can modulate BMP or Wnt signaling pathways are also of pharmacological interest, as these conserved pathways are not only crucial for embryonic development, but are also important in adult homeostasis. Aberrant signaling is linked with several major diseases such as cancer, osteopathies and Alzheimer’s disease. New drug-like lead compounds that target and modulate BMP or Wnt signaling could therefore be of therapeutic utility [18–20].

We carried out a zebrafish-based phenotypic screen for BMP and Wnt signaling modulators using the Strathclyde Natural Products Library as a potential source of novel, bioactive compounds. The library consists of 5000 methanolic plant extracts representing 90% of plant families worldwide. Several active extracts were in this zebrafish screen that mimicked phenotypes previously reported in zebrafish mutants for BMP or Wnt signaling pathway members. Of particular interest was an extract of *Jasminum gilgianum* (Oleaceae), a plant native to Papua New Guinea. As the embryos treated with crude extract displayed ectopic tails, we postulated that a constituent of this plant extract may act as a modulator of BMP and/or non-canonical Wnt signaling [6,7].

 The aim of this study was to isolate the active constituent of *Jasminum gilgianum* responsible for inducing ectopic tail formation and to characterize the compound’s putative modulatory activity on BMP and/or non-canonical Wnt signaling. Our results showed that para-coumaric acid methyl ester is responsible for the bioactivity of *Jasminum gilgianum* and that this compound most likely acts as a modulator of the Wnt/PCP pathway.

## Materials and Methods

### Chemicals and Reagents

Dimethyl sulfoxide (DMSO, 99.9% spectroscopy grade), chloroform and methanol (HPLC grade) were purchased from Acros Organics. Ethylacetate was purchased from ChemLab. Para-coumaric acid methyl ester (pCAME) was purchased from Frinton Laboratories (New Jersey, USA).

### Plant Material

The Strathclyde Natural Products Library (SNPL) was available for this study through collaboration with the Strathclyde Institute for Drug Research (SIDR) at the University of Glasgow and the Scottish Universities Life Science Alliance (SULSA). The SNPL is composed of 5000 methanolic plant extracts, representing 90% of plant families, pre-treated to remove the reactive compounds commonly responsible for false positive results in screening assays (e.g. tannins). The samples, available in 96-well plates, were dissolved in 100% DMSO at a concentration of 10 mg/ml and were kept at -20°C. Prior to use, the plates were thawed and centrifuged at room temperature, and each well was mixed thoroughly by pipetting before an aliquot was taken for screening. Aliquots of methanol-extracted *Jasminum gilgianum* leaves were dissolved in 100% DMSO for zebrafish experiments or in 100% methanol for chromatographic fractionation. 

### Bioassay-Guided Fractionation and Isolation Of Active Constituent

Dry methanolic extract of *Jasminum gilgianum* was separated by thin layer chromatography (TLC) on 0.2 mm Alugram SIL G/UV254 plates (Macherey-Nagel). Ten milligrams of the extract were dissolved in 100 µL methanol and loaded manually onto a TLC plate. Separation was performed in a saturated chamber using chloroform/ethylacetate (80/20). After TLC development, constituents separated as bands were revealed under UV irradiation at 254 and 365 nm, and by spraying the plate with a universal reagent (1% vanillin/H_2_SO_4_). Individual bands of non-sprayed plates were extracted with methanol, evaporated to dryness, and redissolved in DMSO for bioactivity testing (see further).

In order to obtain a larger amount of the active band, preparative column chromatography of 0.2 g methanolic extract of *J. gilgianum* was performed using silica gel (60 mesh, 15 to 40 nm, Merck) with chloroform/ethylacetate (80/20). Fractions of about 2 mL were collected, and the fractions known to contain the active band (as monitored by TLC) were pooled. Several preparative runs yielded 14.4 mg of the fraction.

The constituents present were further separated by HPLC using a LaChrom *Elite* HPLC system (VWR Hitachi) and a 3 µm pentafluorophenyl (PFP, 150 x 4.60 mm, Phenomenex) column. Detection was performed by diode array detection (DAD). The profile of gradient elution was: double-distilled water (ddH_2_O) (A) and methanol (B); 0-15 min, from 50 to 100% B; 15-25 min 100% B; 25-40 min from 100 to 50% B. Separated constituents of repeatedly injections were collected, pooled and dried under nitrogen for bioactivity testing.

### NMR and HRFTMS

The NMR spectral data of the isolated bioactive compound are identical to those reported in the Aldrich Library of 13C and 1H FT NMR Spectra (Pouchert, C. J., and J. Behnke, The Aldrich Library of 13C and 1H FT-NMR Spectra, 75 and 300 MHz, Aldrich Chemical Company, Milwaukee, WI, 1993.) for 3-(4-methoxyphenyl)-2-propenoic acid (*E*-form) also known as p-coumaric acid methyl ester. The isolated compound was elucidated by ^1^H (proton) and 2D HMBC (two-dimensional Heteronuclear Multiple Bond Correlation) NMR spectroscopy using a 400 MHz Jeol NMR system at SIPBS equipped with a 40TH5AT/FG probe. The compound’s molecular formula of C_10_H_10_O_3_ was determined by direct injection on a HRFTMS (high resolution Fourier transform mass spectrometer) using the LTQ-Orbitrap. A commercially available reference standard (Fluka No. 65420) was also used to confirm the structure and chromatographic properties of the sample compound.

### Zebrafish

For all experiments wild-type zebrafish embryos of the AB and transgenic *cmlc2*:eGFP (cardiac myosin light chain 2:eGFP reporter) strains were used. Adult zebrafish were reared under standard aquaculture conditions at 28.5°C on a 14/10 hour light/dark cycle. Embryos were collected after group matings and kept in embryo medium (17 mM NaCl, 0.2 mM KCl, 0.18 mM Ca(NO_3_)_2_, 0.12 mM MgSO_4_, 1.5 mM HEPES buffer pH 7.1-7.3 and 0.6 µM methylene blue). Staging of embryos was done according to Kimmel [21].

### Ethics Statement

All animal procedures were performed in accordance with Belgian and European Laws, guidelines and policies for animal experimentation, housing and care (Belgian Royal Decree of 6 April 2010 and European Directive 2010/63/EU of 20 October 2010 on the protection of animals used for scientific purposes). The Ethics Committee on Animal Experimentation of the University of Leuven has approved this project with the number P101/2010.

### Compound Treatment of Embryos

Phenotypic screening was performed in 24-well plates, and 15 embryos were used per condition in a volume of 1 mL per well. Each condition was tested in duplicate or triplicate. Test solutions were prepared in embryo medium. The DMSO concentration in each well never exceeded 2%. Continuous treatment of embryos was done from the 2- to 4-cell stages up to 48 hours post-fertilization (hpf). Pulse treatment with compound for 1h was performed at tailbud stage (10 hpf) or 1 to 3-somites stages (10.5 - 11 hpf). Embryos were incubated at 28.5°C and dysmorphologies were scored at 24 hpf and 48 hpf. Pictures were taken at 48 hpf, embryos were sedated with 1x tricaine solution (80 µg/mL tricaine in 0.02% w/v sodium phosphate), and methylcellulose was used to embed embryos.

### Whole-Mount in situ Hybridizations (WISH)

Digoxigenin-UTP antisense RNA probes were synthesized using the DIG Labeling Kit (Roche Diagnostics). For WISH analysis, control and compound-treated embryos were fixed at the appropriate developmental stages and were subjected to an *in situ* protocol originally described by Thisse and Thisse [22].

### Microinjections of Morpholino Oligonucleotides

For *jnk2* knockdown experiments, an antisense morpholino oligomer (MO) designed to bind to an exon - intron junction of the zebrafish *jnk2* was synthesized by Gene Tools (Philomath, Oregon). This MO has been described in a previous report [23]. A stock solution of 2 mM was microinjected into the yolk of 1- to 2-cell stages AB embryos using an Eppendorf FemtoJet microinjector. The injected dose was 8 ng per embryo.

### Cells, culture conditions and treatments

Normal human keratinocytes were cultured in keratinocyte-specific medium (Invitrogen). Cells were seeded 24 hours before the experiment in 10 cm^2^ dishes, 10^6^ cells per dish. Cells were treated for 1 or 2 hours with vehicle (0.2% DMSO), SP600125 (20 µM, 10 µM), or pCAME (56 µM, 7 µM, 3.5 µM). Subsequently, anisomycin was added in a final concentration of 10 µg/mL to cell medium. Anisomycin triggers stress-induced apoptosis and thus activates Jnk signaling [24]. 

### Immunoblot

Following 45 minutes of anisomycin treatment, cells were washed twice with ice cold PBS buffer (Invitrogen) and lysates were made with RIPA-buffer (Invitrogen). PhosStop (Roche) phosphatase inhibitor cocktail and c*O*mplete (Roche) protease inhibitor cocktail were added to lysis buffer. Protein concentration was determined using BCA assay (Pierce BCA Protein Assay Kit, Thermoscientific). Samples were prepared in duplicate, loaded in a NuPage Novex 10% Bis-Tris gel (Invitrogen) and subjected to SDS-PAGE. Proteins were transferred to nitrocellulose membrane and probed with C-jun and phosphorylated C-jun (p-C-jun, Ser63) monoclonal antibodies (Cell signaling). Detection was performed using the Odyssey infrared imaging system.

### Imaging

Zebrafish embryos were photographed using a Leica MZ10F stereomicroscope equipped with a DFC310 FX digital camera run by Leica Application Suite software (version 3.6.0). Pictures were processed using Microsoft Powerpoint and Office Picture Manager.

## Results and Discussion

### Zebrafish-Based Screen of Plant Extracts to Identify Bioactive Natural Products

A total of 3160 methanolic plant extracts from the Strathclyde Natural Products Library (SNPL) were screened in zebrafish embryos, with the aim of identifying bioactivities leading to phenotypic defects reminiscent of BMP and Wnt mutants [4–7]. Embryos at the 2- to 4-cell stages were subjected to continuous incubation with 20 and 100 µg/mL of each plant extract. Treated embryos were microscopically scored at 8, 24 and 48 hpf for morphological defects or death. 356 extracts induced embryonic dysmorphologies. These extracts were further assessed in a secondary screen for concentration-dependency and reproducibility of the phenotype. Ten extracts (hits) were selected for further analysis based on their ability to induce BMP- and Wnt-like mutant phenotypes. Interestingly, an extract obtained from *Jasminum gilgianum* (Oleaceae), a plant native to Papua New Guinea, induced tail duplication in zebrafish embryos.

### Bioassay-Guided Isolation of para-Coumaric Acid Methyl Ester (pCAME)

**Figure 1:**
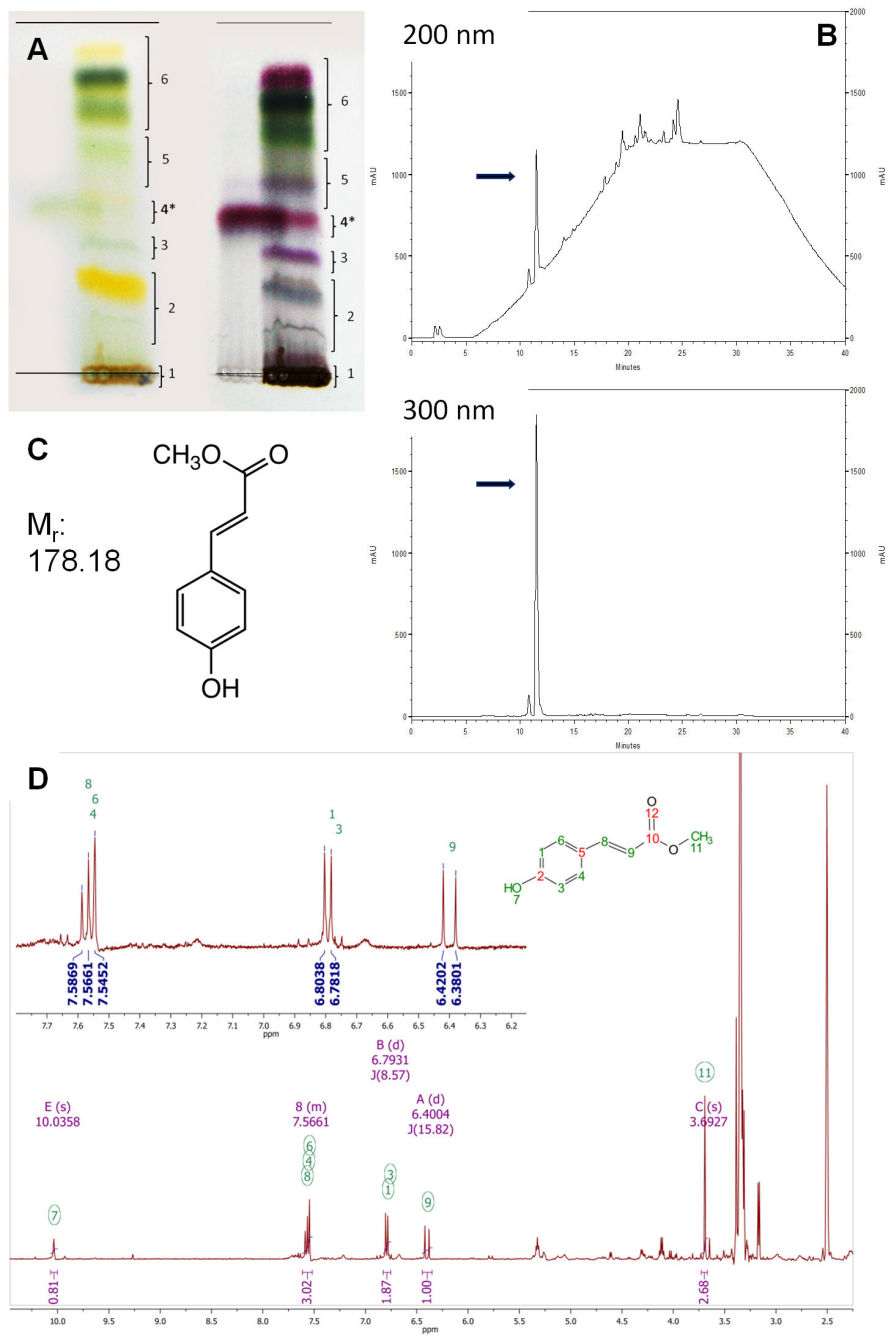
Isolation of pCAME. (A) Typical normal phase TLC separation of the crude methanolic extract of *Jasminum gilgianum*, daylight view (left), plate sprayed with 1% vanillin/H_2_SO4 (right). Black star denotes band of interest. (B) Typical HPLC chromatograms of preparative fraction 4 at 200 and 300 nm, black arrows denote signal due to pCAME. (C) Molecular structure and molecular weight of pCAME. (D) NMR spectral data.

To isolate the active constituent responsible for this activity, bioassay-guided fractionation of the *J. gilgianum* extract was performed. The crude methanolic extract was first separated using silica-based thin layer chromatography. Each band detected was tested on zebrafish embryos, and the tail duplication activity was identified in band 4 (see Figure 1A). Preparative liquid chromatography was then performed to obtain larger amounts of the primary constituents present in this band, which were further separated by HPLC using DAD to monitor the separation process. Each peak detected was collected and tested for activity, thereby identifying the primary peak (see Figure 1B) as inducing the tail duplication phenotype. Identification and structural elucidation of the active compound was performed using HPLC in combination with HRFTMS (high resolution Fourier transform mass spectrometry) and 2D NMR spectroscopy. The NMR spectral data (see Figure 1D) of the isolated biologically active compound from *J. gilgianum* was elucidated as (*E*)-methyl 3-(4-hydroxyphenyl) acrylate also known as para-coumaric acid methyl ester (pCAME) (Figure 1C). The compound’s molecular formula of C_10_H_9_O_3_ for [M-H]^-^ (calc. 177.0557; found 177.0556) was determined by direct injection on an HRFTMS using an LTQ-Orbitrap. A commercially available reference standard was also used to confirm the structure and chromatographic properties of the sample compound. Fortuitously, the compound was commercially available and was purchased from Frinton Laboratories for further experiments. NMR analysis confirmed that the compound provided by the supplier was identical to the active compound isolated from *J. gilgianum*.

### Phenotypic Characterization of pCAME-Treated Embryos

**Figure 2:**
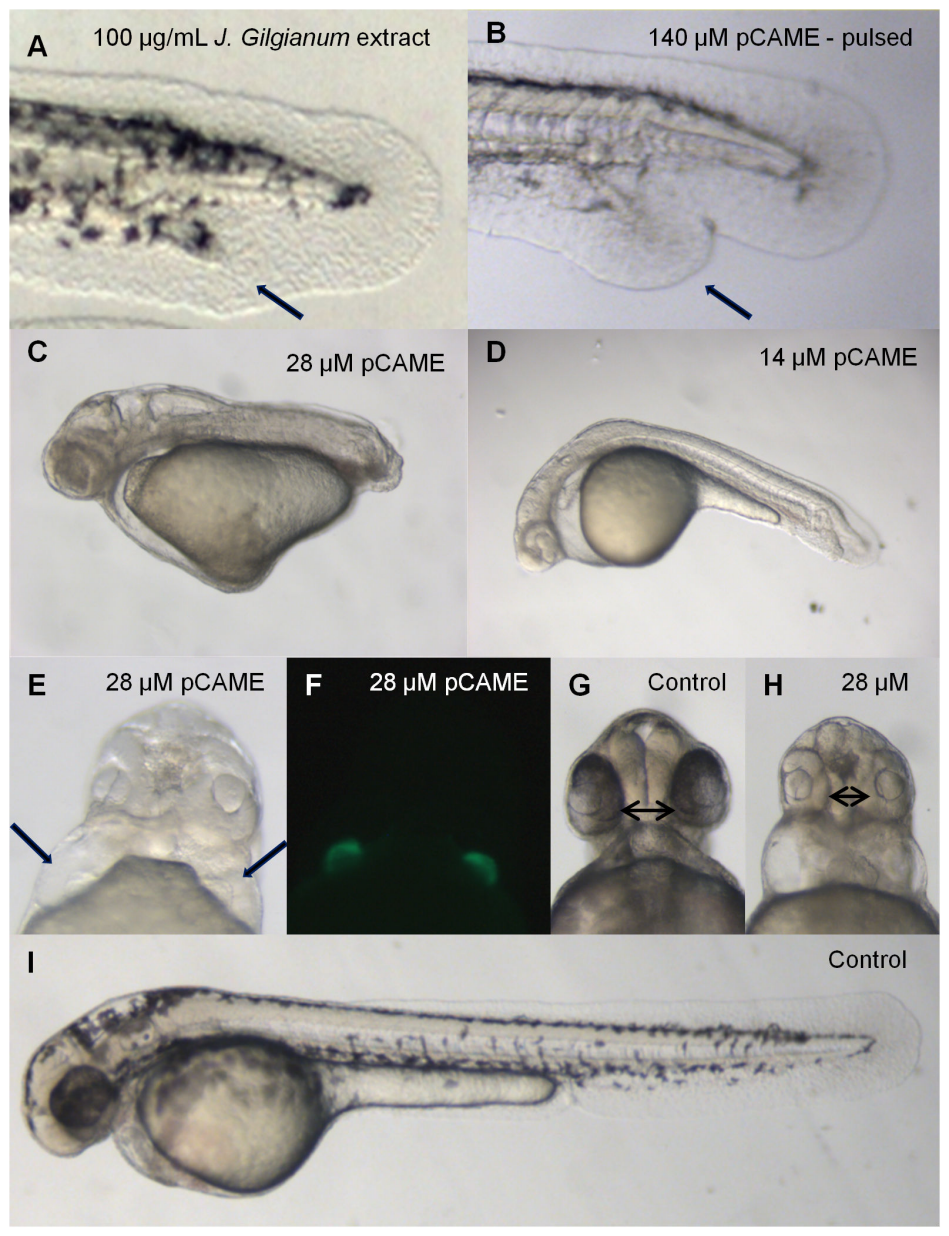
Phenotypic characterization of pCAME-treated embryos. (A) Embryo treated with 100 μg/mL crude methanolic extract of *J. gilgianum*. Black arrow denotes ectopic tail. Unless otherwise noted, all treatments were performed at 2- to 4-cell stages. (B) Embryo treated with 140 μM pCAME pulsed 1h at tailbud stage, black arrow denotes ectopic tail; (C) pCAME-treated embryo with 28 μM and with (D) 14 μM pCAME; (E) Embryo treated with 28 μM pCAME; black arrows denote 2 hearts; (F) *cmlc2*:eGFP embryo treated with 28 μM pCAME; (H) Embryo treated with 28 μM pCAME; black double arrow indicates synophthalmia; (G) and (I) Vehicle-treated control (1% DMSO), black double arrow denotes normal distance between eyes. All embryos are at 48 hpf. The main phenotypic characteristics of pCAME-treated embryos are: tail duplication, AP-axis shortening, absence of pectoral fins, decrease in pigmentation, body curvature, synophthalmia and *cardia* *bifida*.

The crude methanolic extract of *Jasminum gilgianum* (Oleaceae) at a concentration of 100 µg/mL consistently induced tail duplication in treated embryos (Figure 2A). A concentration-response experiment was performed with pure pCAME to analyze in more detail the phenotype/s induced by this compound. Treatment of 2- to 4-cell stages embryos with pCAME resulted in a dose-responsive shortening of the anterior-posterior (AP) axis, (compare 28 µM pCAME (Figure 2C) with 14 µM pCAME-treated embryos (Figure 2D)). Embryos treated with 14 µM pCAME displayed tail duplication, although this phenotype had a variable penetrance, ranging from 7 to 15%. Other dysmorphologies observed at 48 hpf were the absence of pectoral fins, decrease in pigmentation, body curvature, pericardial edema, *cardia bifida* (Figure 2E) and synophthalmia (Figure 2H). Confirmation of the cardiac defects was done using transgenic *cmlc2*:eGFP (cardiac myosin light chain promoter driving eGFP) embryos treated with 28 µM pCAME, in which 100% of treated embryos displayed *cardia bifida* (Figure 2F). In an attempt to induce only the (late) tail duplication phenotype without causing other dysmorphologies due to perturbation of signaling pathways required for early embryonic patterning, a pulse treatment was performed at late tailbud to early somitogenesis stages. Treatment with 140 µM pCAME for 1h at tailbud stage resulted in embryos showing a milder decrease in general pigmentation and tail duplication (Figure 2B) in up to 30% of the embryos. Pulse treatment for 1h at 1- or 3-somite stages for 1h increased the percentage of ectopic tail formation to 67 and 87%, respectively.

### Analysis of Tissue-Specific Marker Expression in pCAME-Induced Ectopic Tails

**Figure 3:**
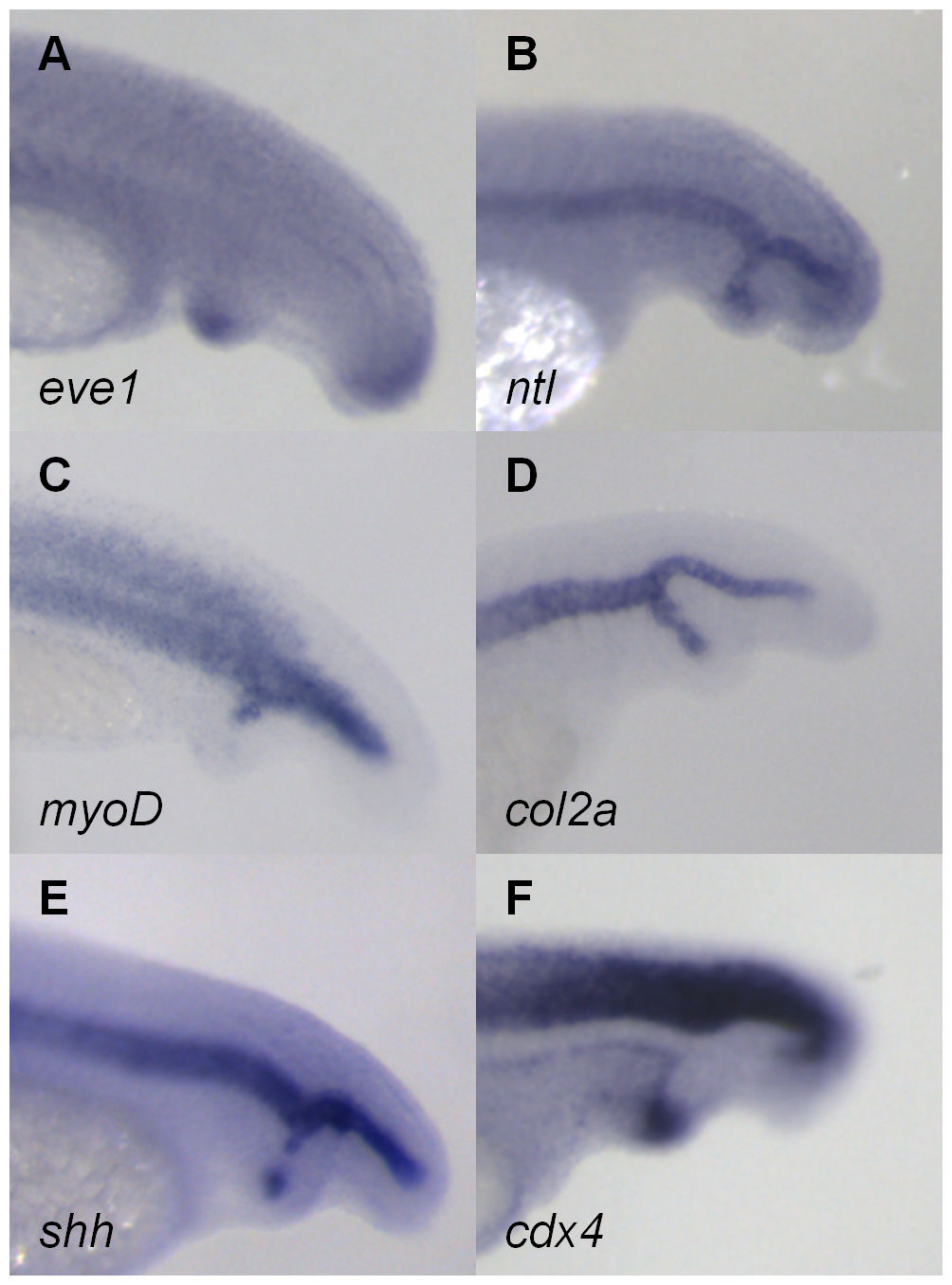
Analysis of tissue-specific marker expression in pCAME-induced ectopic tails. (A) *eve1*; (B) *ntl*; (C) *myoD*; (D) c*ol2a*; (E) *shh*; and (F) *cdx4* expression. All embryos were pulsed with 140 μM pCAME for 1h at tailbud stage and fixed at 30 hpf. Lateral views.

Tail development in zebrafish starts from a pool of undifferentiated cells in the tailbud. During tail protrusion, several signaling pathways – including nodal, fibroblast growth factor, BMP and Wnt pathways – play a concerted role in regulating this process [25,26]. Pyati et al. studied the role of BMP signaling in the ventral and posterior mesoderm during mid- and late-gastrula stages [6], using a transgenic zebrafish line expressing a dominant-negative BMP receptor-GFP fusion protein inducible by heat shock [6]. This study showed that from mid-gastrulation onwards, BMP signaling is important for tail patterning and the inhibition of secondary tail formation. To explore whether the same tissues in pCAME-induced ectopic tails were affected as those previously reported after BMP-DN overexpression, the following markers were used: *myogenic differentiation factor* (*myoD*)*, sonic hedgehog* (*shh*)*, no tail* (*ntl*)*, even-skipped1* (*eve1*)*, collagenase2a* (*col2a*)*, crestin, neurogenin1* (*neurog1*) and *caudal type homeobox transcription* factor *4* (*cdx4*). Embryos were pulsed for 1h at tailbud stage with 140 µM of pCAME and fixed at 30 hpf. The secondary tails expressed both tailbud markers *eve1* (Figure 3A) and *ntl* (Figure 3B). The ectopic tails contained muscle tissue, as marked by *myoD* (Figure 3C) expression. Duplication of the *col2a* (Figure 3D) expression domain proved the presence hypochord cells while *shh* expression (Figure 3E) revealed the presence of notochord cells. Of the neural tube markers (*neurog1* and *cdx4*), only *cdx4* was expressed in the ectopic tails (Figure 3F). Expression of the neural crest marker *crestin* was not observed. Our marker analysis correlated well with those reported by Pyati and colleagues [6]. In summary, the ectopic tails were shown to possess tailbud cells, somites, hypochord and notochord, but lacked neural tissue at 30 hpf.

### pCAME Acts Synergistically with BMP Signaling Inhibitors

Inhibition of BMP and/or non-canonical Wnt signaling can lead to the formation of a secondary tail, according to the model of Yang et al [7]. In this model, both pathways have a distinct function to ensure proper tail morphogenesis. The anteriorward migration of the ventroposterior mesodermal precursors in the tailbud is directed by BMP signaling immediately prior to tail protrusion. These cells disrupt the association of the caudal notochord cells with the yolk while migrating. During this process, non-canonical Wnt signaling increases intracellular adhesion in the caudal notochord cells by influencing the localization of cadherin to the plasma membrane, thereby promoting the association of caudal notochord cells. Ectopic tail formation has been reported to occur in BMP-deficient embryos. A defective migration of the mesodermal progenitors can heavily disturb the caudal notochord cells. Isolation of caudal notochord cells from the rest of the pool during tail protrusion then leads to tail bifurcation. This phenomenon has also been observed in embryos defective in non-canonical Wnt signaling. Due to a lack of sufficient cadherin at the plasma membrane, cohesion is not strong enough to keep the caudal notochord cells together. Again, caudal notochord cells become isolated and thus, form an ectopic tail. The secondary tails are ventrally positioned and contain muscle and notochord tissue. Yang et al. demonstrated that by simultaneously inhibiting both BMP and non-canonical Wnt signaling, the percentage of ectopic tails increases [6,7]. During early gastrula stages, BMP is crucial for dorsoventral patterning of the embryo. An inhibition of BMP at early stages of development results in a phenotypically dorsalized embryo [27]. This phenotype was never observed in pCAME-treated embryos. Therefore, it is not likely that pCAME interferes with early BMP signaling but rather, based on previous reports and our own data, influences non-canonical Wnt/PCP signaling.

Based on these findings, an experiment was carried out using dorsomorphin, a small-molecule inhibitor of the BMP pathway, and its more BMP receptor-selective structural analog LDN-193189 [8,28]. We postulated that if pCAME acts as an inhibitor of non-canonical Wnt signaling, then an increase in the percentage of tail duplications should be seen when pulsed together with BMP inhibitors. Embryos between tailbud and early somitogenesis stages were pulsed for 1h with 70 µM pCAME in the absence or presence of 5 µM or 10 µM dorsomorphin/LDN-193189.

**Figure 4:**
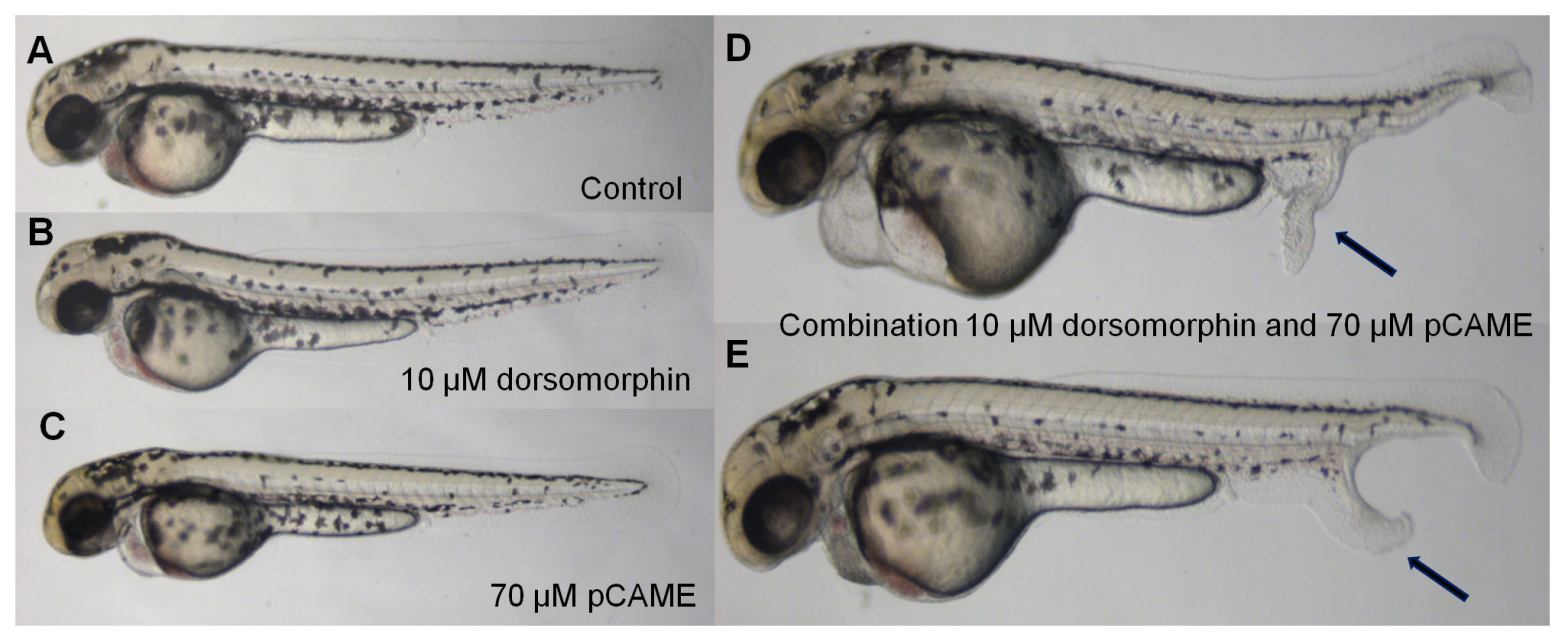
Synergy between pCAME and dorsomorphin. (A) Wild type pulsed with 1% DMSO in Danieau’s solution; (B) 10 µM dorsomorphin pulse; (C) 70 µM pCAME pulse; (D) and (E) Combination of 10 µM dorsomorphin and 70 µM pCAME pulse. Black arrows denote ectopic tails. All AB embryos are at 48 hpf and pulsed for 1h at tailbud stage. A significant increase of the percentage of ectopic tail formation was observed as well as a more pronounced phenotype.

Table 1 represents the result of the 3 combined experiments. Pulsing with subthreshold doses of either 70 µM pCAME, 5 or 10 µM dorsomorphin alone resulted in very few embryos displaying ectopic tails (2.2% for 70 µM pCAME and 10 µM dorsomorphin, and 0% for 5 µM dorsomorphin). However, the pulse combination of 70 µM pCAME with 10 µM dorsomorphin yielded 55.5% ectopic tails, while a pulse combination of 70 µM pCAME with 5 µM dorsomorphin yielded 24.4% ectopic tails. The results of the pulse combination experiment of 70 µM pCAME and 10 µM dorsomorphin are displayed in Figure 4. Thus a significant increase (p<0.001; Fisher’s exact test) in the percentage of ectopic tails was observed when subthreshold doses of pCAME and dorsomorphin were administered in combination. Moreover, the ectopic tails were phenotypically more pronounced. Pulsing with 50 µM dorsomorphin alone yielded a maximum of 25.9% ectopic tails.

**Table 1:** Pulse experiment with pCAME and dorsomorphin.

**Condition**	**Total *n***	**Ectopic tails**	**Synergy Yes/No**	**Fisher’s Exact test P< 0.001**
Control	45	0.0 %	n/a	
pCAME 70 µM	45	2.2 %	n/a	
Dorsomorphin 10 µM	45	2.2 %	n/a	
Dorsomorphin 5 µM	45	0.0 %	n/a	
pCAME 70 µM + Dorsomorphin 10 µM	45	55.5 %	Yes	***
pCAME 70 µM + Dorsomorphin 5 µM	45	24.4 %	Yes	***

Note: Embryos were pulsed for 1h at late gastrula/early somitogenesis (bud to 3-somite stages) and scored at 48 hpf; n/a: not applicable; Table represents data pooled from three independent experiments using (each time) 15 embryos per condition. Fisher’s exact test: Pulse combinations vs. Control condition vs. pCAME condition vs. Dorsomorphin conditions

As more recent findings indicate that dorsomorphin may have additional targets, we also analyzed a more selective BMP inhibitor, LDN-193189 [28]. As shown in Table 2, pulsing with 70 µM pCAME resulted in 6.7% ectopic tails. Pulsing with 5 or 10 µM LDN-193189 did not yield any tail duplications. When we combined the conditions, an increase in ectopic tail formation was observed, and the effect was larger than the combined effects of each compound. The combinations with 70 µM pCAME and 10 µM LDN-193189 yielded 35.6% and the combination with 5 µM LDN-193189 yielded 26.7% ectopic tails. Similar to the results obtained after combined exposure to pCAME and dorsomorphin, a significant increase (p<0.001; Fisher’s exact test) in the percentage of ectopic tails was observed when subthreshold doses of pCAME and LDN-193189 were administered in combination. Pulsing with 80 µM, an 8-fold higher concentration of LDN-193189, yielded a maximum of 23.3% ectopic tails.

**Table 2:** Pulse experiment with pCAME and LDN-193189.

**Condition**	**Total *n***	**Ectopic tails**	**Synergy Yes/No**	**Fisher’s Exact test P< 0.001**
Control	90	0.0%	n/a	
pCAME 70 µM	90	6.7%	n/a	
LDN-193189 10 µM	90	0%	n/a	
LDN-193189 5 µM	90	0%	n/a	
pCAME 70 µM + LDN-193189 10 µM	90	35,6%	Yes	***
pCAME 70 µM + LDN-193189 5 µM	90	26.7%	Yes	***

Note: Embryos were pulsed for 1h at late gastrula/early somitogenesis (bud to 3-somite stages) and scored at 48 hpf; n/a: not applicable; Table represents data pooled from three independent experiments using (each time) 30 embryos per condition.

Fisher’s exact test: Pulse combinations vs. Control condition vs. pCAME condition vs. LDN-193189 conditions

In conclusion, the mechanism by which pCAME induces ectopic tails appears to be synergistic with that of dorsomorphin or LDN-193189. This further supported our hypothesis that pCAME modulates non-canonical Wnt signaling, one of the key signaling pathways important for proper tail morphogenesis.

### Convergence and Extension Defects Induced by pCAME

**Figure 5:**
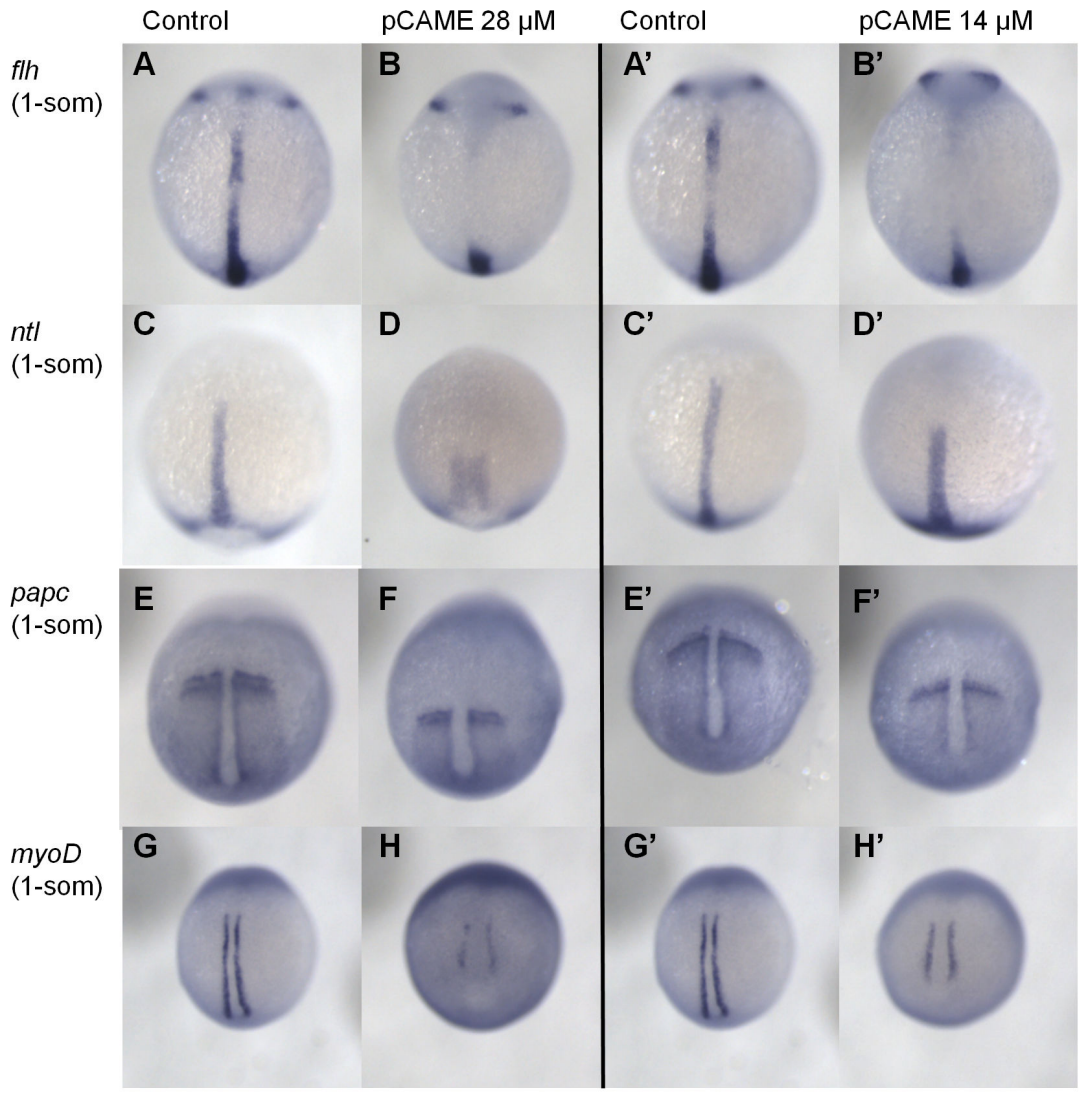
pCAME induces convergence and extension defects. (A) and (B) *flh* expression; (C) and (D) *ntl*; (E) and (F) *papc*; (G) and (H) *myoD*. All embryos were treated at 2-4 cell stages with 14 or 28 μM pCAME and fixed at 1 somite stage. Dorsal view. Expression domains of compound-treated fish are altered as would be predicted for CE defects. Embryos have a broader and shorter notochord and impaired convergence and extension of the para-axial mesoderm and the somites.

Convergence-extension (CE) movements occur during gastrulation, when cells migrate to and intercalate at the dorsal midline and as such, extend the embryo along the anterior-posterior (AP) axis. Both BMP signaling and non-canonical Wnt are important for proper CE movements [29–31]. To visualize CE defects in pCAME-treated embryos, WISH analyses were performed with the following markers: *floating head* (*flh*), *ntl*, *paraxial protocadherin* (*papc*), *myoD* and an *in situ* hybridization probe cocktail of *distal-less homeobox gene 3* (d*lx3*)*, shh, and hatching gland* (*hgg*). Embryos were treated at 2- to 4-cells stages with 14 and 28 µM pCAME and fixed at 1- or 5-somites stages. Compared to wild-type embryos, the compound-treated embryos displayed a wider and shorter notochord, as visualized by *flh* and *ntl* expression (Figure 5A, B, C, D and A’, B’, C’, D’). The edges of the para-axial mesoderm, as marked by *papc*, are farther apart, suggesting CE defects (Figure 5E, F and E’, F’). The somites (*myoD* expression pattern) do not converge all the way to the midline and do not extend anterior-posteriorly as in controls (Figure 5G, H and G’, H’). Furthermore, the expression domain of *dlx3* in the neural plate was expanded both in 14- and 28 µM-treated embryos (data not shown). Overall, the different markers tested provided supporting evidence that CE movements in pCAME- treated embryos were impaired. Thus, pCAME was most likely interfering with one of the pathways that ensure proper CE movements in the gastrulating zebrafish embryo.

### Investigation of C-jun N-terminal Kinase (Jnk) as a Possible Target of pCAME within Wnt/PCP Signaling

Given that structurally-related compounds of pCAME were reported to inhibit both MAP kinases and tyrosine kinases [32,33], we looked for possible kinase targets present in the Wnt/PCP pathway. C-jun N-terminal kinase (Jnk) belongs to the family of MAPKs, and is downstream of the receptor complex of the Wnt/PCP pathway, becoming activated by Rac GTPase [34]. The Jnk family in mammals is encoded by three related genes: *jnk1, jnk2* and *jnk3*. In zebrafish, 4 *jnk* genes have been identified that give rise to the following proteins: Jnk1a-1, Jnk1a-2, Jnk2 and Jnk3. During gastrulation, only transcripts of *jnk1a-1*, *jnk1a2* and *jnk2* are present. Morpholino knockdown of *jnk1a-1* and *jnk1a-2* had no phenotypic effect on zebrafish embryos. Knockdown of *jnk2*, on the other hand, induced severe CE defects and was found to be important for normal CE movements during zebrafish gastrulation [23].

**Figure 6:**
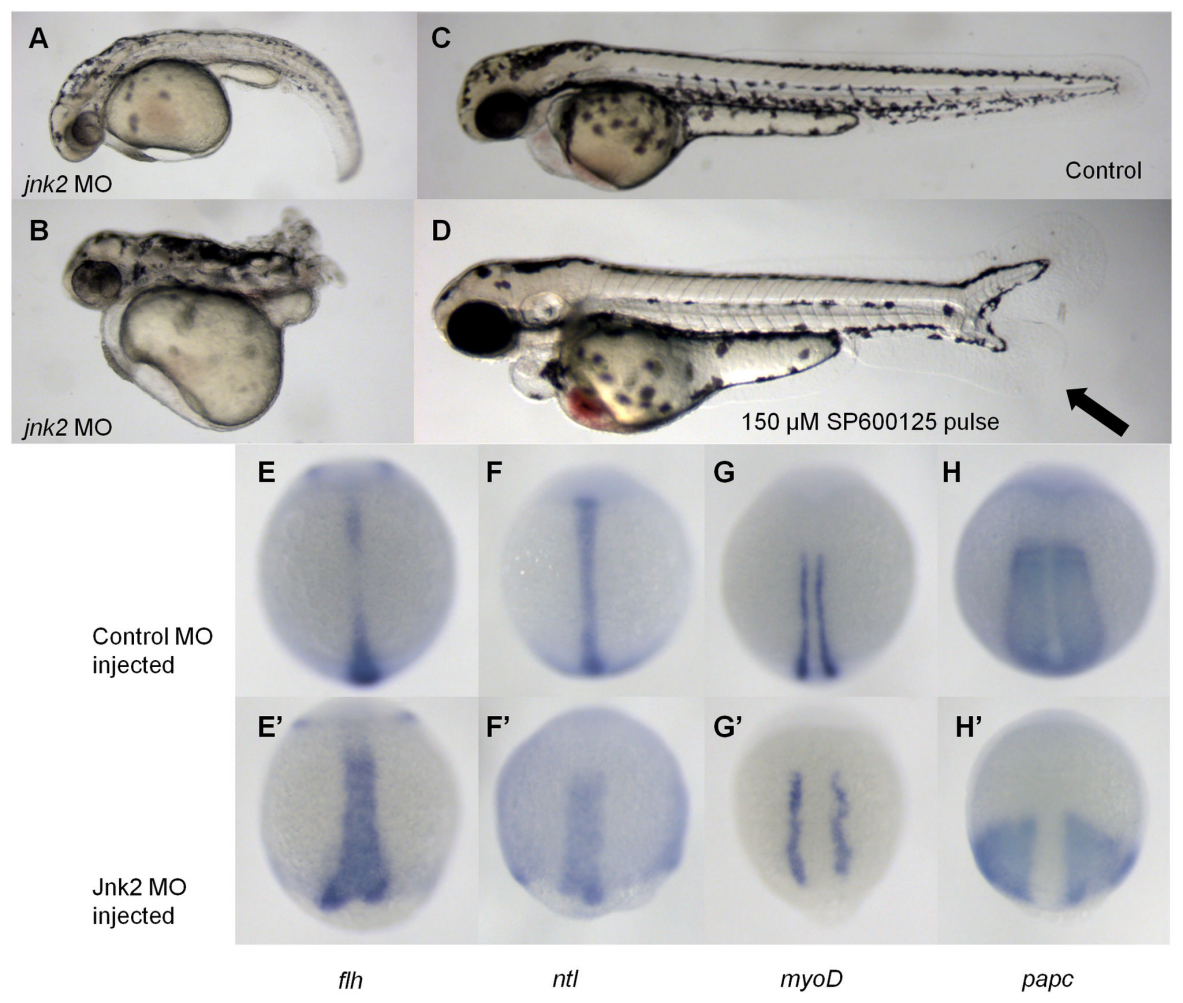
*jnk2* morpholino knockdown induces convergence extension defects. (A) and (B) 2 mM *jnk2* MO-injected fish A: 38 % B: 9 %; (C) Wild type; (D) 150 μM SP600125 pulsed for 1 h at tailbud stage. All embryos are at 48 hpf. Black arrow denotes duplicated tail. (E-H) Control MO injected fish; (E’-H’) *jnk2* MO injected fish; (E) *flh*; (F) *ntl*; (G) *myoD*; (H) *papc*.

In our hands, *jnk2* morpholino knockdown resulted in 38% of injected embryos displaying body curvature (Figure 6A), and 9% of the embryos displaying a complete lack of a tail (Figure 6B). These embryos are reminiscent of 28 µM pCAME-treated embryos (Figure 2C). 53% of injected embryos were normal (Figure 6C), and no tail duplications were observed in these knockdown fish. Notably, however, tail duplication is only induced in a large percentage of embryos after pulse treatment with pCAME at late tailbud to early somitogenesis stages. 

*jnk2* knockdown has previously been shown to impair Wnt/PCP signaling and therefore to cause CE defects in zebrafish embryos [23]. Our results also show the notochord of *jnk2* morpholino-injected fish to be broader and shorter, as visualized by WISH with *flh* and *ntl* (Figure 6E-E’-F-F’). The *myoD* expression pattern indicated that the convergence of the somites was impaired (Figure 6G-G’), while the *papc* expression pattern indicated a failure of the para-axial mesoderm to extend (Figure 6H-H’). In addition, the neural plate was broader than normal, as indicated by *dlx3* expression (data not shown). Together with the previously reported findings [23], these data indicate that knockdown of *jnk2* causes CE defects.

Since it was not possible to test for induction of ectopic tails in *jnk2* MO-injected embryos, we used a known small-molecule inhibitor of Jnk, SP600125, to perform pulsed Jnk2 inhibition experiments. SP600125 was discovered by Bennett et al. as a reversible ATP-competitive inhibitor of Jnk [35–37]. Embryos at tailbud stage were pulsed for 1 hour with different concentrations of SP600125 (200, 150, 100, 50 and 1 µM). Embryos pulsed with 1 and 50 µM appeared normal. At higher concentrations, embryos displayed ectopic tail formation: 30% at 150 and 200 µM; 16.7 % at 100 µM. A representative picture of an embryo pulsed with 150 µM SP600125 is displayed in Figure 6D. 

**Figure 7:**
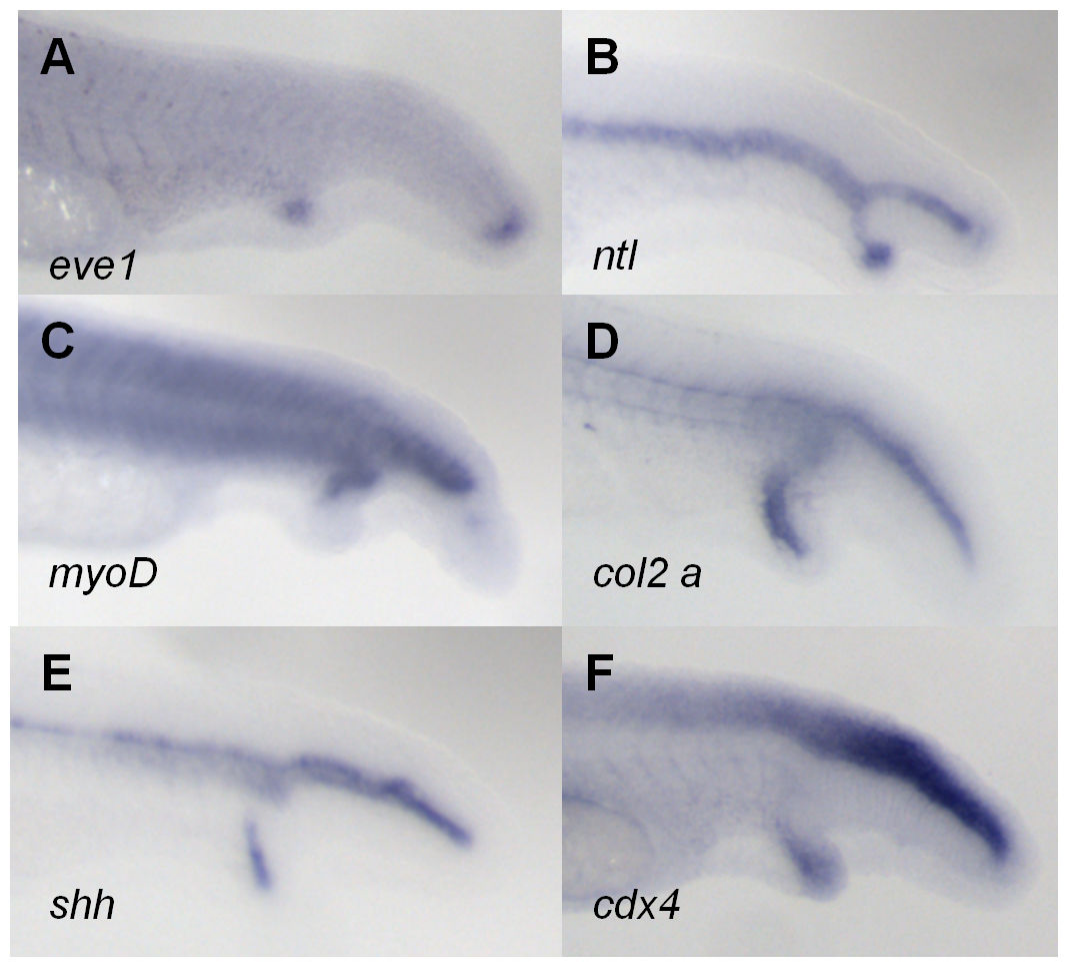
Tissue marker analysis in SP600125-induced ectopic tails. (A) *eve1*; (B) *ntl*; (C) *myoD*; (D) c*ol2a*; (E) *shh*; and (F) c*dx4* expression. All embryos were pulsed with 140 μM pCAME for 1h at tailbud stage and fixed at 30 hpf. Lateral views.

WISH analysis was performed to determine whether the expression pattern of tissue markers in SP600125 ectopic tails resembled that of pCAME-treated embryos. The same markers were used as previously: *myoD, shh, ntl, eve1, col2a, crestin, neurog1* and *cdx4*. Embryos were pulsed for 1h at tailbud stage with 150 µM of SP600125 and fixed at 30 hpf. The ectopic tails possessed tailbud cells (Figure 7A-B), somites (Figure 7C), hypochord (Figure 7D) and notochord (Figure 7E) tissue. From the neural tube markers, only *cdx4* was present in the ectopic tails (Figure 7F). No duplication of the expression domain of *neurog1* and *crestin* was seen. In conclusion, the tissue-marker expression patterns observed in the ectopic tails induced by pulsing with 150 µM SP600125 were identical to those of pCAME-treated embryos.

**Figure 8:**
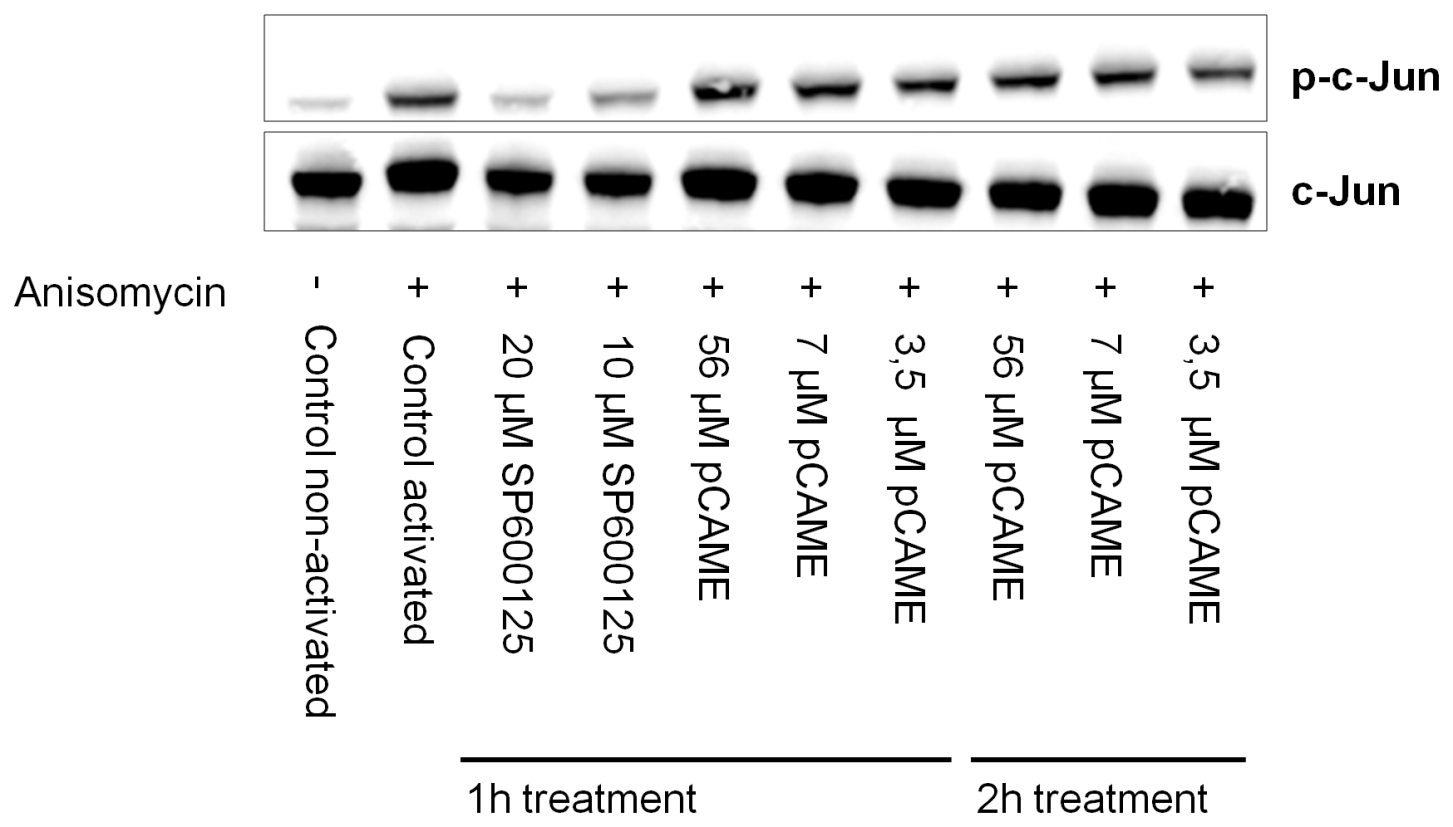
Immunoblot of C-jun and phospho-C-jun. Representative immunoblot of C-jun and phospho-C-jun protein for investigating the inhibitory capacity of pCAME on Jnk. HK cells were treated for 1 or 2 hours with vehicle, SP600125 or pCAME, subsequently activated with anisomycin (10 µg/mL) and lysed.

Based on this data, an *in vitro* experiment was performed to check the inhibitory capacity of pCAME on Jnk kinase activity. Normal human keratinocytes (HK) were treated for 1h or 2h with vehicle, SP600125 or pCAME, subsequently activated with anisomycin (10 µg/mL) and lysed. Upon activation by anisomycin, Jnk phosphorylates its downstream targets, including C-jun. C-jun is a component of the activator protein-1 (AP1) transcription factor [38,39]. After blotting on nitrocellulose membrane, levels of C-jun protein and phosphorylated C-jun (p-C-jun) protein were detected. In the event that Jnk would be inhibited during cell activation, levels of p-C-jun would decrease. Figure 8 displays a representative immunoblot for C-jun and p-C-jun protein. SP600125 displayed an inhibitory effect on both total C-jun and p-C-jun levels in activated HK cells after 1 hour of treatment. In contrast, pCAME did not decrease levels of p-C-jun after 1 hour of treatment. Increasing the treatment time to 2 hours still did not affect p-C-jun levels. 

To summarize, we observed that morpholino knockdown and pharmacological inhibition of Jnk by SP600125, phenocopies pCAME-treated embryos. However, the immunoblotting experiments revealed that pCAME has no effect on C-Jun phosphorylation. Based on this data, pCAME does not appear to inhibit Jnk – at least not directly. Although SP600125 is generally used as specific Jnk inhibitor [35–37], the compound is able to inhibit more kinases then only Jnk isoforms. According to studies of Bain et al., several other protein kinases included in their test panel were inhibited with similar or greater potency by SP600125 [40]. Thus, it is possible that the ectopic tail phenotype we observe may be due to an inhibition of another kinase different from Jnk, kinases farther downstream of Jnk signaling, or even multiple kinases. 

Evidence for a multi-target action of pCAME is based on previous reports describing it as an inhibitor of tyrosinase, an enzyme that is responsible for the production of melanin [41–43]. We performed a comparative test in zebrafish using phenyl thiouracil (PTU), another known tyrosinase inhibitor. Aside from the lack of pigmentation, no pCAME-like dysmorphologies or ectopic tail formation was seen in embryos pulsed with PTU. Therefore we believe that the decrease in pigmentation seen in pCAME-treated embryos is solely due to tyrosinase inhibition, and that this activity is separate from the other phenotypes observed. Moreover, the possibility that pCAME could modulate other pathways essential for zebrafish tail formation such as those of Nodal and Fgf are unlikely as left-right asymmetry defects were never observed in pCAME-treated embryos and the midbrain-hindbrain boundary remained fully intact (data not shown)[44,45].

## Conclusions

This study indicates the usefulness of zebrafish as an *in vivo* bioassay for the identification of novel natural products that inhibit developmental pathways relevant for human disease. In this work, para-coumaric acid methyl ester was isolated from the crude methanolic extract of *Jasminum gilgianum*, a plant native to Papua New Guinea, on the basis of this molecule’s ability to induce ectopic tail formation in zebrafish embryos. These secondary tails were positioned ventrally and have tailbud cells, somites, hypochord and notochord tissue present. No evidence for presence of neural tissue was found. This phenotype is highly reminiscent of the one obtained upon overexpression of a dominant-negative BMP receptor in post-gastrula zebrafish embryos [6] and following inhibition of BMP and/or non-canonical Wnt signaling, according to the model of Yang et al. [7].

According to this model [7], two pathways are involved in ectopic tail formation: BMP and non-canonical Wnt signaling. Both pathways play a role in preventing the caudal notochord cells of the tail bud from sloughing off and forming a secondary tail as tail protrusion proceeds. Our experimental data suggests that pCAME interferes likely with non-canonical Wnt signaling. Furthermore, we observed that SP600125 is able to induce identical ectopic tails. However, the immunoblotting experiments to assess levels of C-Jun and p-C-Jun excluded Jnk as a direct target of pCAME. Therefore, other kinases targeted by SP600125 could also be possible targets of pCAME. Further research will be required to identify this compound’s precise mechanism of action. 
